# Detection of cancers three years prior to diagnosis using plasma cell-free DNA

**DOI:** 10.1158/2159-8290.CD-25-0375

**Published:** 2025-09-04

**Authors:** Yuxuan Wang, Corinne E. Joshu, Samuel D. Curtis, Christopher Douville, Vernon A. Burk, Meng Ru, Maria Popoli, Janine Ptak, Lisa Dobbyn, Natalie Silliman, Josef Coresh, Eric Boerwinkle, Anna Prizment, Chetan Bettegowda, Kenneth W. Kinzler, Nickolas Papadopoulos, Elizabeth A. Platz, Bert Vogelstein

**Affiliations:** 1 Sidney Kimmel Comprehensive Cancer Center, Johns Hopkins University School of Medicine, Baltimore, MD; 2 The Ludwig Center for Cancer Genetics and Therapeutics, Baltimore, MD; 3 The Sol Goldman Pancreatic Cancer Research Center, Johns Hopkins University School of Medicine, Baltimore, MD; 4 Department of Epidemiology, Johns Hopkins Bloomberg School of Public Health, Baltimore, MD; 5 Department of Pharmacology and Molecular Sciences, Johns Hopkins University School of Medicine, Baltimore, MD; 6 Division of Quantitative Sciences, Johns Hopkins University School of Medicine, Baltimore, MD; 7 The Howard Hughes Medical Institute, Baltimore, MD; 8 Department of Medicine, NYU Grossman School of Medicine, New York, NY; 9 Department of Population Health, NYU Grossman School of Medicine, New York, NY; 10 School of Public Health, The University of Texas Health Science Center at Houston, Houston, TX; 11 Department of Laboratory Medicine and Pathology, University of Minnesota, Minneapolis, MN; 12 Department of Neurosurgery, Johns Hopkins University School of Medicine, Baltimore, MD

## Abstract

To explore how early can cancers be detected prior to clinical signs or symptoms, we assessed prospectively collected serial plasma samples from the Atherosclerosis Risk in Communities (ARIC) study, including 26 participants diagnosed with cancer and 26 matched controls. At the index time point, eight of these 52 participants scored positively with a multicancer early detection (MCED) test. All eight participants were diagnosed with cancer within 4 months after blood collection. In six of these 8 participants, we were able to assess an earlier plasma sample collected 3.1 to 3.5 years prior to clinical diagnosis. In four of these six participants, the same mutations detected by the MCED test could be identified, but at 8.6 to 79-fold lower mutant allele fractions. These results demonstrate that it is possible to detect circulating tumor DNA more than three years prior to clinical diagnosis, and provide benchmark sensitivities required for this purpose.

## INTRODUCTION

Despite recent advances in cancer prevention and treatment, cancer remains a leading cause of death in the United States(1). Though the interval between tumor initiation and metastasis is on the order of decades (2–4), many cancers are detected late in the course of disease, and such cancers account for the vast majority of cancer deaths. An effective earlier detection strategy to identify subclinical cancers could lead to the delivery of treatments when they are most effective and most likely to be curative (5–8).

Circulating tumor DNA (ctDNA) is one of the most promising biomarkers for earlier detection(9–26). Prospective trials using ctDNA in multicancer early detection (MCED) tests have been encouraging, though with limited sensitivities for early-stage disease (27,28). Moreover, the natural course of disease was changed by the results of these studies because they were interventional - participants who scored positively in the test were subsequently evaluated for cancer by imaging or other diagnostic modalities, and if they had cancers, they were immediately treated. It is thus impossible to know whether or when these participants would have been diagnosed with cancer in the absence of the test.

We had an opportunity to perform a proof-of-principle study that could address these challenging issues. Here, we evaluated the ability of an MCED test to detect early cancers in prospectively collected plasma samples from the Atherosclerosis Risk in Communities (ARIC) study (29). Though prospective, this study was not interventional. Thus, the natural course of disease in these participants was recorded and reported, including cancer diagnoses months or years after the samples were collected.

## RESULTS

### Phase I: Evaluation of Early Plasma Samples using a Targeted Driver Gene Panel

**Figure 1:**
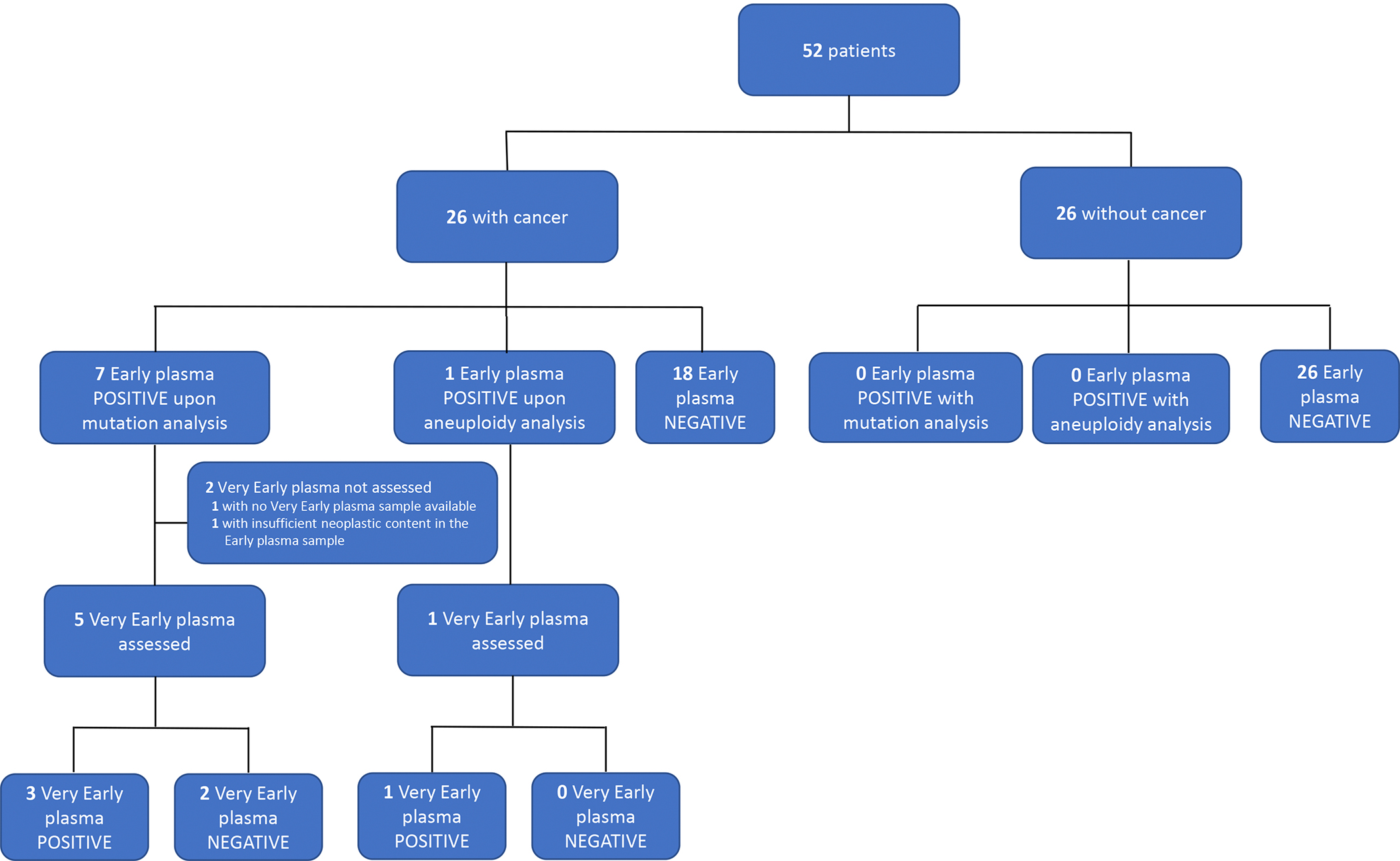
Participants included in study and ctDNA analysis results.

In Phase I of this study, blood samples from 52 ARIC participants were accessed, including 26 individuals with cancers diagnosed within 6 months of blood collection and 26 individuals without cancer as controls individually matched for age, race, and sex. The controls were individuals who did not have a history of cancer and did not develop cancer within a follow-up period ranging from 17.1 to 19.6 years. The clinical status of these participants was blinded to the investigators performing tests on the cell-free DNA (cfDNA) from their plasma. Hereinafter, these plasma samples will be referred to as “Early plasma samples.” For mutation detection in purified DNA from the Early plasma samples, a driver gene panel consisting of 121 commonly mutated regions in a total of 40 driver genes was evaluated (Supplementary Table S1). The assessment was made using a technique that implemented error reduction strategies including independent sequencing of both strands of DNA(30). In all but one participant (Methods), mutations identified in the plasma could be evaluated in DNA from the matched leukocytes to exclude germline mutations as well as mutations arising from clonal hematopoiesis of indeterminate potential (CHIP)(31–33). A median of 6123 template DNA molecules per mutation from the leukocytes were assessed to ensure the absence of CHIP (Supplementary Table S2). Based on assessment of approximately 5 mL of plasma per participant, seven participants were found to have at least one detectable mutation (range: 1 to 3 mutations; Supplementary Table S2). All seven individuals who scored positively in this test were diagnosed with cancer within 0.4 to 3.7 months after the blood collection, while none of the 26 controls scored positively in the same test (Fig. 1).

A total of fifteen mutations were detected in these seven participants, with mutant allele frequencies (MAFs) ranging from 0.27% to 11.2% (Supplementary Table S2). Although tumor tissue was not available for assessment, the mutations detected were characteristic of the diagnosed tumor types (Supplementary Table S2). *APC*, *KRAS*, *PIK3CA* or *TP53* mutations were detected in the three participants with colorectal cancers; *KRAS* and *TP53* mutations were detected in the participant with pancreatic cancer; a *TP53* mutation was diagnosed in the participant with breast cancer; and *TP53* and *KRAS* mutations were detected in the participant with lung cancer.

### Phase II: Evaluation of Very Early Plasma Samples using the Targeted Driver Gene Panel

Six of the seven positive participants had an available plasma sample (hereinafter referred to as “Very Early plasma samples”) collected 3.1 to 3.5 years prior to the diagnosis of cancer at a prior ARIC study visit (Supplementary Tables 1 and S3). DNA from these Very Early plasma samples was purified and used to determine whether ctDNA was detectable using the driver gene panel of amplicons (Supplementary Table S1). Two of the 6 participants were positive in Very Early plasma sample, and the only mutations identified were identical to those observed in the Early plasma samples (Supplementary Table S2). In INDIA 2225, a participant who was later diagnosed with colon cancer, three mutations were detected in the Early Plasma, and all three were also detected in the Very Early plasma sample. The MAFs were ~11 to 23-fold lower in the Very Early plasma sample than in the Early plasma sample (Supplementary Table S2). In INDIA 2228, two mutations were detected in the Early plasma, and one of these two mutations was detected in the Very Early plasma sample, albeit at 115-fold lower frequency (11.2% vs 0.097%; Supplementary Table S2).

### Re-analysis of Early Plasma Samples using whole genome sequencing of cell-free DNA

As noted above, the driver gene panel used for mutation detection in the Early plasma revealed 1 to 3 mutations in each of the seven positive participants (Supplementary Table S2). To get a more accurate estimate of the mutation burden in these participants, we searched for additional mutations in the following way. First, we performed deep (70–100X) whole genome sequencing (WGS) on the Early plasma samples as well as 20x WGS of matched leukocytes (Methods). From these data, we selected up to 96 mutations in the plasma DNA that were absent in the leukocyte DNA for each participant. These mutations were carefully chosen based on the quality of the sequencing data and other criteria designed to minimize errors in subsequent analysis (Methods). Note that virtually all of the chosen mutations were passengers rather than drivers, but were apparently clonal because they were selected on the basis of high mutant allele frequencies in the Early plasma samples (Methods). Because WGS data contains many more errors than obtained with duplex sequencing methods such as SaferSeqS (30), we expected some of the selected mutations to be artifacts of library preparation or sequencing rather than genuine somatic mutations derived from neoplastic cells. Germline variants can also be confused with somatic mutations when the sequencing depth of the leukocytes at a particular position is low (Methods). To overcome these obstacles, we designed primers for each of the selected mutations, thereby creating a personalized mutation panel of up to 96 mutations per participant, and validated them by SaferSeqS – identically as described above for the driver gene panel.

We used the personalized mutation panel to assess six of the seven participants who scored positively in the Early plasma samples using the targeted driver gene panel and had an available Very Early plasma sample (Supplementary Table S4). The MAF of the seventh participant (INDIA 2210) was only 0.33%, making this strategy infeasible because of the cost – it would require far more than 100-fold depth to confidently choose 96 mutations from WGS data on a sample with such a low MAF. In the other six participants, we considered a mutation from the personalized panel to be *bona fide* if it were present in the plasma sample but absent in the leukocyte DNA samples when assessed by SaferSeqS. We were thereby able to identify a median of 39 *bona fide* mutations per participant (range: 16 to 94) in Early plasma samples in the six participants evaluated (Supplementary Tables S4 and S5). This represented a 10 to 46-fold increase in the number of mutations that could be confidently scored compared to the targeted driver gene panel, in which a median of 2 mutations per participant were detected (Supplementary Table S4). The median MAF of the mutations scored with the personalized panel was similar to, but slightly higher than, the median MAF identified with the driver gene panel (3.84 vs. 2.67%; Supplementary Table S4).

While the above evaluation was performed for cases who tested positive in their Early plasma sample using the targeted driver gene panel, the targeted panel of amplicons is not expected to detect tumor-specific mutations in all cancer participants. This is either because some cancers do not have mutations in the queried amplicons, or they do not shed sufficient amounts of tumor DNA into the circulation. We sought to address the first of these possibilities by assessing aneuploidy through the low-depth WGS of DNA from the Early plasma samples. While methods for the detection of ctDNA using aneuploidy is not as sensitive as those using somatic mutations, they require lower DNA input for evaluation and is therefore less affected by the limiting amounts of available plasma in the cohort(34). Accordingly, we performed low-depth (1x coverage) WGS of cfDNA from the 45 participants who had no detectable mutations in their Early plasma samples with the targeted driver mutation panel (Fig. 1). Of them, we found one participant to have detectable aneuploidy (INDIA 2207). After unblinding, the participant proved to have cancer diagnosed 2.8 months after the Early plasma sample acquisition. We next performed high-depth WGS on the Early plasma sample as described above, and through comparison to the WGS data on leukocytes, were able to design a personalized panel consisting of 19 mutations for the participant. The average MAF based on the personalized panel was 2.04% in the Early plasma sample, roughly consistent with the estimated tumor fraction of 3.1% based on aneuploidy analysis (Supplementary Table S4). In total, eight participants have a positive Early plasma sample: seven detected with the targeted panel and one with aneuploidy analysis (Fig. 1). The cancer types and stage for all 26 patients in the study are listed in Supplementary Table S5.

### Re-analysis of Very Early Plasma Samples using whole genome sequencing of cell-free DNA

The greater number of mutations identified with the WGS-based personalized mutation panel allowed us to re-assess the Very Early plasma samples for the participants who had a positive Early plasma sample to determine if higher sensitivity could be achieved (Supplementary Table 1). In the two participants (INDIA 2225 and 2228) in whom mutations were identified in the Very Early plasma with the targeted driver gene panel, mutations could also be identified with the personalized panel. In INDIA 2225, 30 mutations were detected in the Early plasma sample and 24 of these 30 (80%) mutations were detected in the Very Early plasma sample (Supplementary Table S4). The MAF decreased by 50-fold in the Very Early plasma samples compared to the Early plasma sample (0.05% vs. 2.5%). In INDIA 2228, 92 mutations were detected in the Early plasma sample and 90 of these 92 (98%) were detected in the Very Early plasma sample (Supplementary Table S4). The MAF decreased by 79-fold in the Very Early plasma samples compared to the Early plasma sample (0.33% vs. 26.3%).

There were four participants in whom mutations could be detected in the Early plasma sample but not the Very Early plasma sample using the targeted driver gene panel (Supplementary Table S4). Using the personalized panel, we were thereby able to identify mutations in the Very Early Plasma sample in one of these four participants (INDIA 2233). In INDIA 2233, 21 mutations were detected in the Early plasma sample and 4 of these 21 (19%) mutations were detected in the Very Early plasma sample (Supplementary Table S4). The MAF decreased by 9-fold in the Very Early plasma samples compared to the Early plasma sample (0.16% vs. 1.39%).

Finally for the participant (INDIA 2207) who had no mutations identified using the targeted driver gene panel but had detectable aneuploidy in the Early plasma sample as described above, 12 of the 19 (63%) mutations identified in the Early plasma sample with the personalized panel were also identified in the Very Early plasma sample (Supplementary Tables S4 and S6). The MAF was 40-fold less in the Very Early plasma sample compared to the Early plasma sample (0.05% vs. 2.04%).

## DISCUSSION

Using a nested case-control study within a community-based cohort in which blood was collected at multiple study visits months before the cancer diagnosis, we demonstrated that a MCED strategy could detect ctDNA in a subset of the cases and none of the controls. Further, we showed that in the majority of the positive cases with an available earlier plasma for assessment, ctDNA could be detected more than three years prior to cancer diagnosis. This pilot study provides benchmark sensitivities required for ctDNA-based MCED tests, with key findings summarized below.

### Early plasma samples.

We identified eight participants (among 26 cancer cases and 26 matched controls) who scored positively based on detectable tumor DNA using the assays described above (Fig. 1 and Supplementary Table 1). All of these eight participants developed cancer within four months after the Early plasma sample acquisition for a sensitivity of 31% (8 of 26; 95% Confidence Interval (CI): 17 to 50%). Three of these cases had colon cancer, and one each had pancreatic, rectal, lung, breast or liver cancer. None of these cases had been diagnosed with cancer, and none had signs or symptoms based on study cohort records specifically suggesting a cancerous state, prior to the Early plasma sample acquisition (Supplementary Table S3). Though the stage of cancer at diagnosis was not available in the cohort study records for five of the participants, the remaining three participants each had Stage I, II, or III cancers (Supplementary Table S3). Five of the participants eventually died from their cancers, while the other three did not (Supplementary Table S3). None of the 26 controls (i.e. those who did not have a history of cancer and did not develop cancer within a follow-up period ranging from 17.1 to 19.6 years) scored positively in these assays.

### Very Early plasma samples.

**Figure 2:**
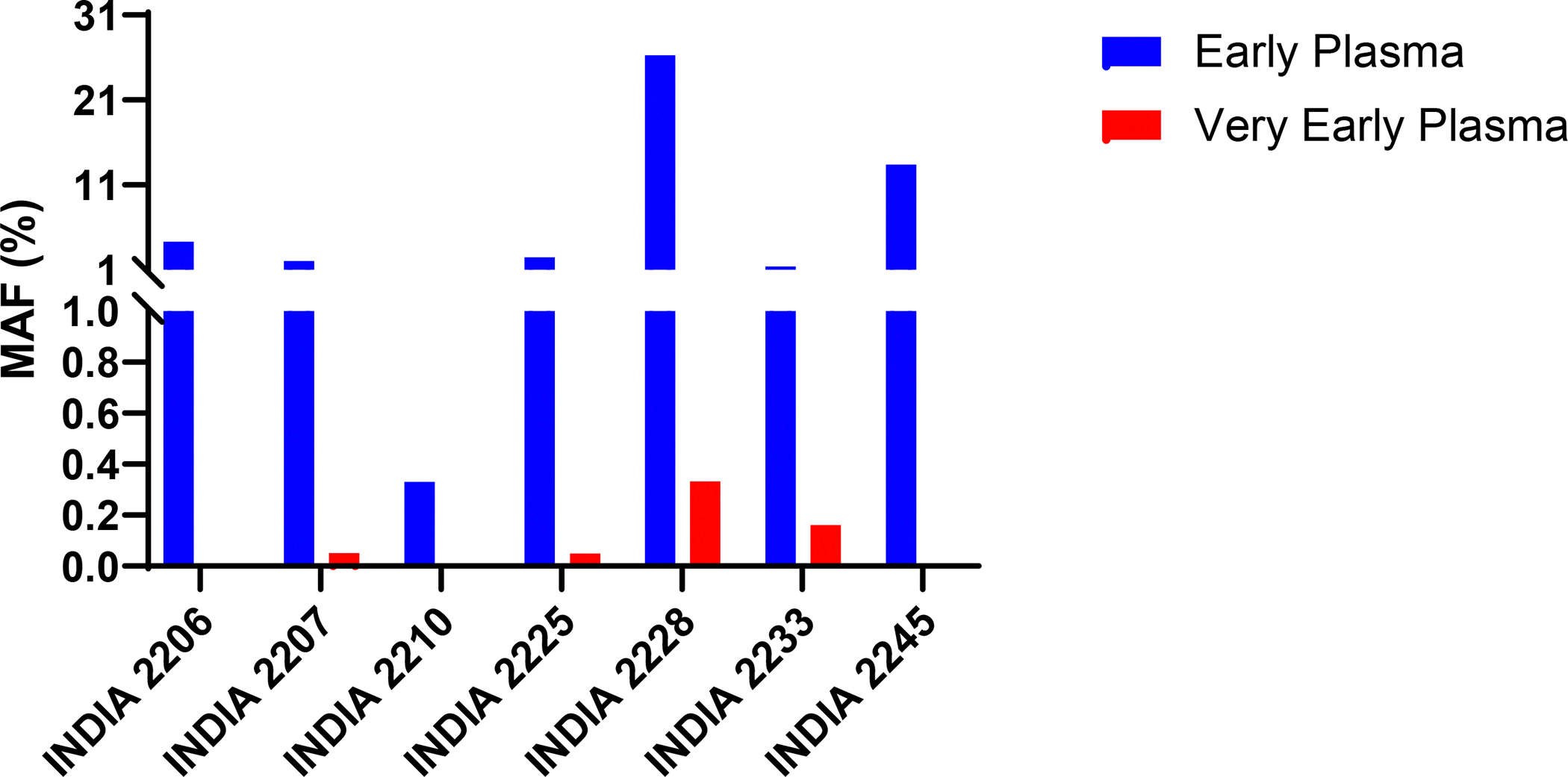
Mutant allele fractions (MAFs) of Early and Very Early plasma samples. Circulating tumor DNA assays were performed on both samples for seven participants. No mutations were detected in the Very Early plasma samples of INDIA 2206, 2210 and 2245.

For a highly quantitative and sensitive assessment of mutations in cfDNA, we were able to design a personalized mutation panel to assess the Very Early plasma samples from six of the eight participants whose Early plasma samples scored positively (Fig. 1 and Table 1). Note that these personalized mutation panels did not require DNA from the primary tumors of the participants, as primary tumor DNA was unavailable from all cases. The number of mutations that were assessed in the Very Early plasma samples varied from 3 to 94 (Supplementary Table S4). Somatic mutations could be detected in the Very early plasma samples from 4 of the 6 participants (67%, Table 1 and Supplementary Table S4). Notably, the MAFs of mutations in the Very Early Plasma samples were a median of 45-fold lower than those of the Early plasma samples (Table 1 and Fig. 2).

### Strengths and Weaknesses.

The unique part of this study was the ability to evaluate plasma samples collected more than three years prior to cancer diagnosis and before any specific signs or symptoms leading to such diagnoses. Another strength of the study was the design of an approach to detect very rare mutations with high specificity in the absence of DNA from the primary tumor. The high sensitivity and specificity of this approach are due to the careful selection of the mutations to be assessed (see Methods), the relatively large number of mutations assessed, the high conversion efficiency of original DNA to library DNA and the independent sequencing of both the Watson and Crick strands of the DNA (“duplex sequencing”)(30). Approaches using duplex sequencing have high sensitivity to detect mutations when DNA from tumors are available, and these approaches are generally referred to as “bespoke” (30,35,36). We extend such technologies by showing that bespoke or personalized assays can be designed based on high depth sequencing of plasma DNA. At present, this approach is applicable to plasma samples in which the MAF is >0.5%. As sequencing costs decrease and sequencing accuracy increases in the future, this approach should be applicable to participants with lower MAFs in their plasma.

A weakness of this pilot study is that we could evaluate only seven participants at the Very Early plasma time points. Nevertheless, the finding that ctDNA could be detected in *any* of the participants three years prior to cancer diagnosis provides new and important insights into the timeline and sensitivity required for a successful MCED test. Large prospective cohorts, like ARIC, that have multiple blood collections over long term from participants with subsequent cancer diagnoses are unique. And it may be challenging to reproduce cohorts like this in the future because there is such a great interest in interventional studies assessing ctDNA. Such interventions preclude knowledge of the natural history of these participants with respect to their development of cancer, as noted in the Introduction.

Our sensitivity for detecting cancers up to 6 months prior to diagnosis was ~31% (8 of 26 participants with cancer). This is similar to the sensitivity of detection of cancers in the prospective and interventional studies that have been performed to date [11, 12]. The sensitivities and specificities will almost always be lower in prospective studies, in which blood was collected prior to cancer diagnosis, than in case-control studies, in which participants already have established cancer diagnoses and thus higher tumor burdens at the time of blood collections. Efforts to increase this sensitivity will be important to improve the earlier detection of cancer. One way is to evaluate a larger volume of plasma, as only 5 mL of plasma was available for assessment in our study. In prior studies, we used at least 10-mL of plasma, an increase which is important for detecting low disease burdens (27,37). Evaluation of much more plasma, using a new generation of plasma collection methods, could be useful in this regard (38). Combining mutational analysis and aneuploidy with other analytes, such as proteomics, methylation, RNA, or fragmentomics could also lead to higher sensitivity for early detection – though at the potential expense of reduced specificity(39–41).

The focus of our study was not to demonstrate the performance of a new MCED test, but rather to demonstrate how early ctDNA can be detected by *any* MCED test. As such, it is worth nothing that the cancer types in the 26 cases in this pilot study do not represent their prevalence in the general population. Although we used a highly specific test that resulted in no false positives in this study, the incidence of cancer in the average risk population is much lower than it is in our cohort. Validation in larger cohorts that better mimic real world cancer prevalence and the target population is necessary to access the performance of the test for MCED. Furthermore, additional studies are necessary to help determine the proper follow-up of a positive result from an ultrasensitive MCED test. Prior studies have demonstrated the utility of a full body imaging and serial blood testing as potential strategies(27). High specificity of any such test is essential to avoid unnecessary anxiety and workup associated with false positives.

Finally, this study establishes the first benchmark for what it would take to actually detect cancers at very early time points. Current technologies for early detection in case control or conventional prospective studies aim for various limits of detection, and these limits are often challenging to define if they are based on machine learning algorithms. But regardless of the technology chosen, this study documents that the detection of cancers three years or more prior to diagnosis will require ~50-fold higher sensitivity than required to detect cancer six months prior to diagnosis. Establishing technologies for achieving this benchmark is a worthy future goal.

## MATERIALS AND METHODS

### Study Population

ARIC (RRID: SCR_021769), a multicenter population-based prospective cohort study of atherosclerosis, was initiated in 1987–1989 when 15,792 mostly White and Black men and women volunteers aged 45 to 64 years were recruited from 4 communities: suburban Minneapolis (MN), Forsyth County (NC), Jackson (MS), and Washington County (MD)-(42). The ARIC participants provided written informed consent. The Institutional Review Boards at each study site approved the ARIC study protocol, and the research was conducted under the U.S. Common Rule. After baseline, participants attended multiple study visits that included interviews, blood collections, and clinical examinations. Cancer diagnoses were ascertained through 2012 by linkage with state cancer registries in the four ARIC states, which was supplemented by abstraction of medical records and routinely collected hospital discharge summaries for cases who were self-reported at a study visit or on an annual or semi-annual follow-up telephone calls(43). Deaths due to cancer as the underlying cause were ascertained through 2012 from death certificates.

Our study was approved by the ARIC committee in 2016 as a two-phase pilot study. Among participants with blood collected at Visit 3 (1993–1995) and who never had a prior cancer diagnosis, we identified participants subsequently diagnosed within 5 years (1998–2000) with a primary cancer of one and only one of 8 sites: breast, colon, rectal, pancreatic, lung, liver, stomach, and esophageal. Participants were eligible to be selected as a control if they had blood collected at Visit 3, never had a cancer diagnosis before or after blood draw (up to 19 years after), and did not die of cancer. Controls were individually matched to cases on age, sex, race (White or Black), ARIC study center, and blood collection year. A total of 247 pairs were identified. Of these, 50 pairs for which the case’s diagnosis was the closest in time after blood collection (within 6 months) were selected for the first phase of the pilot study. Only 26 cases had a sufficient volume to provide 5 mL of filtered or citrated plasma with at least one aliquot remaining in the repository. In total, 26 case-control pairs were available for the study described here. Additional clinical information prior to the Visit 3 blood collection, and prior to and about the cancer diagnosis was abstracted from hospital discharge summaries and medical claims codes. Plasma samples analyzed in this study were prospectively collected in Visit 2, which occurred approximately three years prior to visit 3, and Visit 3 (1993–1995), so meets the definition of a prospective study, even though the samples were not analyzed until years after they were collected. The samples were termed “Very early” and “Early” plasma samples, for Visits 2 and 3, respectively. The ARIC study protocol was approved by the Institutional Review Boards (IRB) of each of the participating centers and informed consent was obtained.

All ctDNA analysis were done in a blinded fashion. Only the clinical information, including case and control status, for the participants who scored positively in the plasma-based assays used were provided to the investigators performing these assays. Investigators performing cfDNA assays remain blinded to the case-control status of the participants who tested negative in this study.

### Blood sample collection and DNA purification

Five mL of plasma, either filtered to remove platelets or citrated, and 3.3 ug of germline DNA from white blood cells (leukocytes) were provided for each participant from Visit 3. For participants who scored positively using the Visit 3 plasma (“Early Plasma” sample), 5 mL of plasma from Visit 2 (1990–1992, “Very Early Plasma” sample) was subsequently provided for all participants except for INDIA 2215. Plasma was purified using BioChain cfDNA Extraction Kit (BioChain, cat #K5011610) using the manufacturer’s recommended protocol. cfDNA was quantified using qPCR using Sso Advanced SYBR Green Supermix (BioRad Cat # 1725271) as directed by the manufacturer and employing the following primers: 5’-CACACAGGAAACAGCTATGACCATGGGTAACAGCTTTATCTATTGACATTATGC-3’ and 5’-CGACGTAAAACGACGGCCAGTNNNNNNNNNNNNNNAAACTTCATGCTTCATCTAGTCAGC-3’. National Institute of Standards and Technology (NIST) human DNA quantification standard NIST SRM 2372a, diluted to 1 ng/ml, served as the reference standard. 2.5 μL of cfDNA or NIST 2372a DNA was added to 97.5 μL of 1:1,000 SYBR Green I diluted in 1X PBS. Amplification and fluorescence detection conditions were as follows: one cycle of 98°C for 120 seconds and then 30 cycles of 98°C for 10 seconds, 57°C for 120 seconds, and 72°C for 120 seconds.

### Library preparation and targeted sequencing

SaferSeqS libraries were made from plasma and leukocytes as previously described (30) with the following modifications. KAPA HiFi HotStart ReadyMix (Roche) was used to amplify the DNA following ligations using the following conditions: 98 °C for 45 s, followed by 8 cycles of 98 °C for 15 s, 60 °C for 30 s and 72 °C for 30 seconds. The amplified DNA was cleaned-up using 1.8 X SPRI beads and eluted in 100 uL EB buffer (Qiagen). The leukocyte DNA was sheared to an average size of ~200bp using a Hielscher UIP400MTP sonicator prior to library preparation. Ten uL of eluted DNA was used per well for targeted enrichment using multiplex, anchored hemi-nested PCR. Targeted sequencing was done as previously described on an Illumina NovaSeq 6000 instrument or a Complete Genomics T7 instrument (30).

### Whole genome sequencing and mutation selection for personalized mutation panels

Whole genome sequencing (WGS) was performed on an Illumina NovaSeq 6000 instrument or a Complete Genomics T7 instrument to a depth of 70–100 X for Early plasma samples, and ~30X for matched normal leukocytes. FASTQ files were generated using Illumina’s bcl2fastq or by Complete Genomic’s Ztron Lite Server. Adapter sequences were removed with Cutadapt (44). The trimmed sequences were then aligned to hg38 reference genome with BWA-MEM with default settings (45). Duplicate sequencing clusters were removed with Picard (http://broadinstitute.github.io/picard). Variants in the plasma sample were called using Strelka2 (Illumina) using the same participant’s leukocytes as the matched normal. Any mutation present in the matched leukocytes, which was available for all participants except for INDIA 2245, were excluded from further analysis. For INDIA 2245, the Very Early plasma was used as the matched normal, as its neoplastic content is expected to be very low. The Very Early plasma sample could definitively exclude the possibility that we were confusing germline variants or other germline artifacts with somatic mutations. However, we could not definitively exclude the possibility that all 16 somatic mutations identified in the Early plasma sample were the result of clonal hematopoiesis of indeterminate potential (CHIP)(31–33), though none of these 16 mutations were detected in the Very Early Plasma sample.

Up to 96 candidate, tumor-specific mutations per participant were selected after excluding mutations in repetitive regions, regions with difficult alignments to the reference genome (hg38), regions that were difficult to amplify efficiently, regions containing single nucleotide polymorphisms, or transitions at CpG sites. These exclusions were informed through prior analysis of samples from individuals without cancer, using whole genome or targeted sequencing. The candidate mutations described above were then used to design a personalized assay (“personalized mutation panel”). For each of the candidate mutations chosen, primers were designed as described previously (30). Primers for all candidate mutations were combined into a single tube for each participant. A hemi-nested, two-stage PCR protocol was used to amplify the regions containing the candidate mutations as described previously(30), except that KAPA HiFi HotStart polymerase was used for amplification ReadyMix (Roche, Indianapolis, IN; cat # KR0370). Following sequencing on a NovaSeq 6000 instrument, the data were evaluated as described(30).

### Mutation Analysis

For the targeted mutation panel, mutation calls were made as previously described (30). Briefly, mutations are present in both the Watson and Crick strands, absent in the matched normal, and classified as oncogenic are considered to be positive. Oncogenic mutations include hotspots in driver gene mutations (46), and inactivating mutations in tumor suppressors genes (2). A positive call is based on the presence of two or more distinct mutant molecules in the targeted panel. For mutations to be considered a *bona fide* mutation in the WGS-derived personalized panels, the mutation had to be present in both the Watson and Crick strands in the DNA, absent in the matched leukocytes when assessed at very high depth, and absent in the plasma sample of an unrelated control without cancer. The same multiplex assay was subsequently applied to the Early and Very Early plasma samples from each participant. Although approximately 96 mutations were assessed in every DNA sample from every participant, only the *bona fide* mutations among the candidate mutations were scored, which are listed in Supplementary Table S6. A positive call is based on the presence of a single mutant molecule in the personalized panel.

### Analysis of Copy Number Alterations

ichorCNA90 version 0.3.2 was used for estimation of tumor fractions (47). Wig files were generated using readCounter with arguments –window 5000000 –quality 30. CreatePanelOfNormals.R was used to generate a panel of normals (n = 124). Parameters tuned for samples with low tumor content (early stage disease) were used. Tumor fraction parameters were initialized at low fractions as follows: --normal “c(0.95, 0.99, 0.995, 0.999)”, the ploidy initialization parameter was 2 --ploidy “c(2)”, estimation of subclonal copy number vents were turned to FALSE --estimateScPrevalence FALSE --scStates “c().” Only calls on the autosomal chromosomes were generated by --chrs “c(1:22)” --chrTrain “c(1:22)”. A threshold of 0.03 (3%) was used as the threshold(47).

### Statistical Analysis

Sensitivity was approximated using the Wilson score interval(48).

## Supplementary Material

1

## Figures and Tables

**Table 1:** Summary of participants in whom Early Plasma sample scored positively.

Participant	Cancer	Stage	Days to cancer diagnosis, Early Plasma	MAF, Early Plasma	Days to cancer diagnosis, Very Early Plasma	MAF, Very Early Plasma	Ratio of MAF in Early Plasma to Very Early Plasma

INDIA 2206	Rectal	III	96	4.31%	1208	Mutation not detectable	Not applicable
INDIA 2207	Liver	Unknown	84	2.04%	1230	0.05%	39.7
INDIA 2210	Colon	II	104	0.33%	1270	Mutation not detectable	Not applicable
INDIA 2215	Pancreatic	Unknown	20	3.37%	Sample not available	Sample not available	Sample not available
INDIA 2225	Colon	Unknown	30	2.50%	1117	0.05%	50.0
INDIA 2228	Lung	Unknown	112	26.26%	1197	0.33%	79.2
INDIA 2233	Colon	I	12	1.39%	1117	0.16%	8.6
INDIA 2245	Breast	Unknown	47	13.40%	1139	Mutation not detectable	Not applicable

MAF = Mutant Allele Frequency

## Data Availability

The sequencing data generated during the study is deposited in the database of EU Genome–Phenome Archive under accession code EGAS00001008068.

## STATEMENT OF SIGNIFICANCE

Earlier detection is a promising strategy to reduce cancer mortality. For cancers of all stages, therapies are more effective with lower disease burden. Here we demonstrate that ctDNA is detectable more than three years before cancer diagnosis and provide estimates for the sensitivity required to achieve such very early detection.
